# Supplementary material for: eDNA-stimulated cell dispersion from Caulobacter crescentus biofilms upon oxygen limitation is dependent on a toxin–antitoxin system
Source: eLife. 2023 Jan 19;12:e80808. doi: 10.7554/eLife.80808 (PMC9851616; doi:10.7554/eLife.80808)
Supplement: Supplementary file 1. [file elife-80808-supp1.docx]

**Supplementary File 1: Plasmids and strains**

|  | **Description / Construction** | **Source / Reference** |
| --- | --- | --- |
| **Strains** | |  |
| YB135 | CB15 WT | (Poindexter, 1964) |
| YB4789 | CB15 WT::miniTn*7*-*gfp* | (Berne *et al.*, 2010) |
| YB4788 | CB15 WT::miniTn*7*-*dsred* | (Berne *et al.*, 2010) |
| AS110 | CB15 WT::miniTn*7*-*gfp* | (Entcheva-Dimitrov & Spormann, 2004) |
| AS109 | CB15 WT::miniTn*7*-*dsred* | (Entcheva-Dimitrov & Spormann, 2004) |
| YB5253 | CB15 ∆*parDE_4_*::miniTn*7*-*dsred* | This study |
| YB5254 | CB15 ∆*parDE_4_*::miniTn*7*-*dsred* | This study |
| FC304 | CB15 ∆*parDE_1_* | (Fiebig *et al.*, 2010) |
| FC975 | CB15 ∆Ψ*parDE_2_* | (Fiebig *et al.*, 2010) |
| FC922 | CB15 ∆*parDE_3_* | (Fiebig *et al.*, 2010) |
| FC915 | CB15 ∆*parDE_4_* | (Fiebig *et al.*, 2010) |
| FC917 | CB15 ∆*relBE_1_* | (Fiebig *et al.*, 2010) |
| FC993 | CB15 ∆*relBE_2_* | (Fiebig *et al.*, 2010) |
| FC980 | CB15 ∆*relBE_3_* | (Fiebig *et al.*, 2010) |
| FC977 | CB15 ∆*relBE_4_* | (Fiebig *et al.*, 2010) |
|  | CB15 ∆*parDE* | A. Fiebig |
|  | CB15 ∆*relBE* | A. Fiebig |
|  | CB15 ∆*parDE relBE* | A. Fiebig |
| FC878 | CB15 ∆*parE_4_* | (Fiebig *et al.*, 2010) |
| YB9497 | CB15 ∆*parDE_4_* ∆*hfsDAB* | This study |
| YB8243 | CB15 WT pRKlac290 | (Berne *et al.*, 2018) |
| YB7040 | CB15 WT pRKlac290-P*parDE_4_* | This study |
| YB9699 | CB15 WT pRKlac290-P*ccoN* | This study |
| YB9495 | CB15 WT::miniTn*7*-*dsred* pRKlac290-P*ccoN* | This study |
| YB9496 | CB15 WT::miniTn*7*-*dsred* pRKlac290-P*parDE_4_* | This study |
| YB5264 | CB15 WT pMT686 | This study |
| YB5265 | CB15 WT pMT686-*parD_4_* | (Fiebig *et al.*, 2010) |
| YB5266 | CB15 ∆*parE_4_* pMT686 | This study |
| YB5267 | CB15 ∆*parE_4_* pMT686-*parD_4_* | This study |
| YB5282 | CB15 WT pMR10-P*ccoN*-*mcherry* | This study |
| YB4779 | CB15 WT pMR20-P*parDE_4_*-*gfp* | This study |
| YB9491 | CB15 WT pMR20-P*parDE_4_*-*gfp* pMR10-P*ccoN*-*mcherry* | This study |
|  |  |  |
| **Plasmids** | |  |
| pRKlac290 | plasmid for *lacZ* transcriptional fusions | (Gober & Shapiro, 1992) |
| pRKlac290-P*parDE_4_* | *parDE_4_* promoter region cloned into pRKlac290 | This study |
| pRKlac290-P*ccoN* | *ccoN* promoter region cloned into pRKlac290 | This study |
| pNPTS138-*hfsDAB* | Non replicative plasmid used for in-frame deletion of the *hfsDAB* operon | (Hardy *et al.*, 2010) |
| pMT686 | Low copy replicating plasmid, xylose inducible, Cm resistant | (Thanbichler *et al.*, 2007) |
| pMT686-*parD_4_* | *parD_4_* under control of the xylose promoter in pMT686 | (Fiebig *et al.*, 2010) |
| pMR20 | Replicating plasmid, IPTG inducible (constitutive in *C. crescentus*) (Tet resistant) | R. Roberts |
| pMR20-*gfp* | *gfp* expression under the control of promoter of choice (YB2137) | M. Lawler |
| pMR20-P*parDE_4_*-*gfp* | *gfp* expression under the control of the *parDE_4_* promoter | This study |
| pMR10 | Replicating plasmid, IPTG inducible (constitutive in *C. crescentus*) (Kn resistant) | R. Roberts |
| pMR10-*mcherry* | *mCherry* expression under the control of promoter of choice (YB4819) | J. Javens |
| pMR10-P*ccoN*-*mcherry* | *mCherry* expression under the control of the *ccoN* promoter | This study |

**References**

**Berne C**, Kysela DT & Brun YV (2010) A bacterial extracellular DNA inhibits settling of motile progeny cells within a biofilm. *Molecular Microbiology* **77**: 815–829.

**Berne C**, Ellison CK, Agarwal R, Severin GB, Fiebig A, Morton III RI, Waters CM & Brun YV (2018) Feedback regulation of *Caulobacter crescentus* holdfast synthesis by flagellum assembly via the holdfast inhibitor HfiA. *Molecular Microbiology* **110**: 219-238.

**Entcheva-Dimitrov P** & Spormann AM (2004) Dynamics and control of biofilms of the oligotrophic bacterium *Caulobacter crescentus*. *Journal of Bacteriology* **186**: 8254-8266.

**Fiebig A,** Castro Rojas CM, Siegal-Gaskins D & Crosson S (2010) Interaction specificity, toxicity and regulation of a paralogous set of ParE/RelE-family toxin-antitoxin systems. *Molecular Microbiology* **77**: 236-251.

**Gober JW** & Shapiro L (1992) A developmentally regulated *Caulobacter* flagellar promoter is activated by 3'enhancer and IHF binding elements. *Molecular Biology of the Cell* **3**: 913-926.

**Hardy GG**, Allen RC, Toh E, Long M, Brown PJ, Cole‐Tobian JL & Brun YV (2010) A localized multimeric anchor attaches the *Caulobacter* holdfast to the cell pole. *Molecular Microbiology* **76**: 409-427.

**Poindexter JS** (1964) Biological Properties and Classification of the *Caulobacter* Group. *Bacteriology Reviews* **28**: 231-295.

**Thanbichler M**, Iniesta AA & Shapiro L (2007) A comprehensive set of plasmids for vanillate-and xylose-inducible gene expression in *Caulobacter crescentus*. *Nucleic Acids Research* **35**: e137-e137.
