# Supplementary material for: eDNA-stimulated cell dispersion from Caulobacter crescentus biofilms upon oxygen limitation is dependent on a toxin–antitoxin system
Source: eLife. 2023 Jan 19;12:e80808. doi: 10.7554/eLife.80808 (PMC9851616; doi:10.7554/eLife.80808)
Supplement: Supplementary file 2. [file elife-80808-supp2.docx]

**Supplementary File 2: Primers used in this study**

| **Sequence (5' → 3')** | **Restriction site (bold sequence)** | **Use** |
| --- | --- | --- |
| AA**CTGCAG**AACGTCGCGCCGGTTTCGGCGA | PstI | P*parDE_4_* cloning into pRKlac290 |
| CCC**AAGCTT**GGGATAGATGTCAAAGGCGGGGG | HindIII |  |
| TT**CTGCA**GTTGGCGCAGCGCT | PstI | P*ccoN* cloning into pRKlac290 |
| TCC**AAGCTT**CTTCTCGCCATC | HindIII |  |
| CGA**CTGCTG**CAGGGTTGAGTGCGATTTC | PstI | P*parDE_4_* cloning into pMR20-*gfp* |
| TCGAT**GGATCC**TTTAAACATTCATCG | BamHI |  |
| GG**GAATTC**CGTTGCGCGGTTGGCGCAGCGC | EcoRI | P*parDE_4_* cloning into pMR10-*mcherry* |
| GG**GACGTC**AGATGGCGAGAAGCGCTCCGGA | AatII |  |
| GCAGCTCTATGCGATCAACA | N/A | *rpoD* amplification (for qPCR) |
| TGTCGTTCTCGACGAACTTG | N/A |  |
| TGGCCGACGATCTTCTACTT | N/A | *ccoN* amplification (for qPCR) |
| AACAGCTGATAGCCCCAGAA | N/A |  |
